# Lung Cancer Stem Cells—Origin, Diagnostic Techniques and Perspective for Therapies

**DOI:** 10.3390/cancers13122996

**Published:** 2021-06-15

**Authors:** Agata Raniszewska, Iwona Kwiecień, Elżbieta Rutkowska, Piotr Rzepecki, Joanna Domagała-Kulawik

**Affiliations:** 1 Laboratory of Hematology and Flow Cytometry, Department of Internal Medicine and Hematology, Military Institute of Medicine, 04-141 Warsaw, Poland; ikwiecien@wim.mil.pl (I.K.); erutkowska@wim.mil.pl (E.R.); 2 Department of Internal Medicine and Hematology, Military Institute of Medicine, 04-141 Warsaw, Poland; przepecki@wim.mil.pl; 3 Department of Internal Medicine, Pulmonary Diseases and Allergy, Medical University of Warsaw, Banacha 1a Street, 02-097 Warsaw, Poland; domagalakulawik@gmail.com

**Keywords:** non-small lung cancer, cancer stem cells, liquid biopsy, immunotherapy, aging, biopsy

## Abstract

**Simple Summary:**

Lung cancer is still a serious oncological problem worldwide. Thus, the biology of this cancer is of interest. Cancer stem cells (CSCs) are involved in tumor initiation and progression. Spontaneously occurring mutations accumulate in stem cells or/and progenitor cells throughout a person’s lifetime resulting in the formation of CSCs. In this review, we discuss the CSC hypothesis with an emphasis on age-associated changes that govern carcinogenesis. The evidence from the scientific literature, as well as our own results and observations, has been presented.

**Abstract:**

Lung cancer remains one of the most aggressive solid tumors with an overall poor prognosis. Molecular studies carried out on lung tumors during treatment have shown the phenomenon of clonal evolution, thereby promoting the occurrence of a temporal heterogeneity of the tumor. Therefore, the biology of lung cancer is interesting. Cancer stem cells (CSCs) are involved in tumor initiation and metastasis. Aging is still the most important risk factor for lung cancer development. Spontaneously occurring mutations accumulate in normal stem cells or/and progenitor cells by human life resulting in the formation of CSCs. Deepening knowledge of these complex processes and improving early recognition and markers of predictive value are of utmost importance. In this paper, we discuss the CSC hypothesis with an emphasis on age-related changes that initiate carcinogenesis. We analyze the current literature in the field, describe our own experience in CSC investigation and discuss the technical challenges with special emphasis on liquid biopsy.

## 1. Introduction

Lung cancer is still a leading cause of cancer-related deaths globally among men (23% of all cancer-related deaths) and women (22% of all cancer-related deaths) [1]. Most people diagnosed with lung cancer are 65 or older; a very small number of people diagnosed are younger than 45. The average age at the time of diagnosis is approximately 70 years [1]. The biggest risk factor for lung cancer is smoking. Worldwide, cigarette smoking alone accounts for over 80% of all lung cancer cases [2]. Other factors, such as air pollution, emission fuel combustion indoor, environmental exposure to radon and asbestos, contribute to the development of lung cancer [3]. As all these risk factors can be prevented through smoking cessation and air purification activities, it is possible to diminish lung cancer incidence and mortality through population-based preventive strategies [2].

Lung cancer is categorized into two main histological types: small cell lung carcinoma (SCLC, 15% of all lung cancers) and non-small cell lung carcinoma (NSCLC, 85% of all lung cancers). NSCLC comprises main histological subtypes: adenocarcinoma (ADC), squamous cell carcinoma (SQCLC) and large cell carcinoma (LCC) [4]. The collected data suggest that lung cancer is a group of histologically and molecularly heterogeneous disorders even within the same histologic subtype [5].

Most NSCLC patients are diagnosed at advanced stage, when the various treatments cannot be curative. Diagnosing lung cancer at the earliest stage is strongly associated with improved survival. Thus, this requires greater readiness of primary care physicians to carefully screen patients at high risk, even with non-specific symptoms [2]. In the course of the disease, lung cancer spreads when cells detach from a tumor and pass through the bloodstream or the lymph vessels to distant areas of the body and grow. This process is metastasis. The most common sites of the spread of lung cancer are the: lymph nodes, liver, bones, brain, adrenal glands [6].

## 2. Lung Cancer and Aging

Metastatic solid tumors, such as lung cancer, remain largely incurable despite improvements in cancer therapies. Aging is still the most important risk factor for lung cancer development. Spontaneously occurring mutations accumulate in somatic cells throughout a person’s lifetime. Most of these mutations do not have a remarkable effect, but some of them can modify key cellular functions. The mutated genes are engaged in tumor biology including: the resistance to cell death, deregulation of metabolism, maintaining cellular proliferation, evading growth suppressors, unstoppable replicative immortality, invasion activation metastasis, inducing angiogenesis genomic instability and mutations [7]. The rates of different mutational processes vary among tumors and cancer types. Although numbers vary widely, most cancers have 1000 to 20,000 somatic point mutations and a few to thousands of insertions, deletions, and rearrangements. Tumors caused by exposure to mutagens, such as NSCLC, present the highest rates of mutations [8]. NSCLC is a heterogeneous illness with unique combinations of somatic molecular changes in individual patients, as well as significant differences in populations around the world with respect to mutation spectra and frequencies. The Cancer Genome Atlas (TCGA) has conducted comprehensive genome studies of NSCLC, displaying a great diversity of molecular variations. In each case of cancer, especially ADC, one molecular alteration dominates, and the term “driver” mutation is used and determinates possible effectiveness targeted therapy [9]. A frequency of driver mutations in Caucasians is: KRAS 15–30%, FGFR1 20%, EGFR 10–30%, ALK 3–7%, BRAF 1–3%, PIK3CA 1–3%, AKT1 1%, HER2 2–5%, MEK1 1%, NRAS 1%, RET 1%, ROS1 1% [10]. Interestingly, it has been proven that aging is tightly associated with developing EGFR mutation in lung cancer [11,12]. Molecular characterization of NSCLC has experienced a big breakthrough in recent years leading to the development of accepted and new targeted therapies (Table 1).

**Figure 1:**
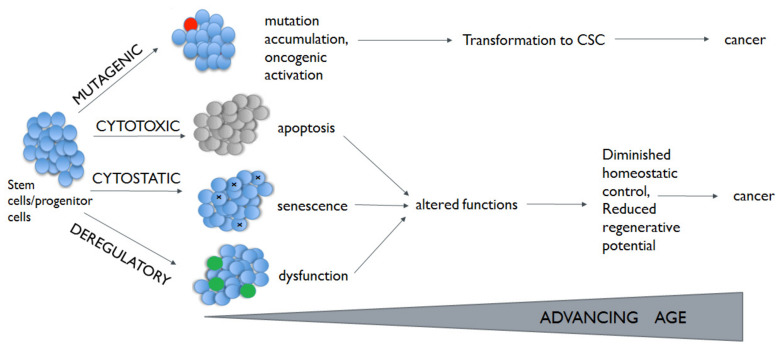
Age-related DNA damage in stem cells. Mutation accumulation and/or oncogenic activation leads to transformation to cancer stem cells (CSCs; marked red) and the initiation of cancer. Cytotoxic and cytostatic effects lead to stem cell senescence or apoptosis. It may result in reduction in the stem cell population. Deregulatory mechanisms lead to reduced homeostatic control and diminished regenerative potential.

At the current stage of knowledge, nine hallmarks of aging are proposed, i.e., genome instability, abrasion of telomere, epigenetic alteration, loss of proteostasis, deregulation of nutrient sensing, damage of mitochondria, cellular senescence, changed intercellular communication and stem cell depletion [28]. One explanation favors the entity of pleiotropic genes, which have opposite effects at different stages; they are beneficial at early stages but are unfavorable at a later age. Thus far, the p53, mammalian target of rapamycin (mTOR) pathway and Wnt signaling have been proposed as antagonistic pleiotropic programmers [29,30]. Alterations in these pathways have been described in different chronic lung diseases, including lung cancer [31,32,33]. Hallmarks of aging in lung cancer are presented in Table 2. Another explanation for aging is the “disposable soma” theory. It claims that organisms age because of an evolutionary trade-off between reproduction, growth, and DNA repair maintenance [34]. While some types of genomic lesions can directly regulate cellular behavior, the effect of DNA damage on the physiology of tissues is mostly believed to be assigned to the cellular response. Participation of stem cells in this process is highly probable. Depending on the nature and damage size, cytostatic, cytotoxic, or mutagenic lesions arising in stem cells have the potential to lead to cells senescence, apoptosis, or transformation (Figure 1). What is more, normal stem cells can become cancer stem cells (CSCs) through the acquisition of mutation and genetic or epigenetic alterations [35] (Figure 1).

Some author suggests that driver mutations increase the cancer cell stemness, even if it is not possible to be said unequivocally that a cell with a higher number of driver mutations corresponds to less uninvolved phenotype of the cells as in the case of normal stem cells [36,37]. There are some data confirming the presence of driver mutations in CSCs (e.g., KRAS in colorectal CSCs [38]). A study performed by Klevebring et al. nicely shows that the vast majority of mutations are shared between CSCs and the bulk primary tumor [39]. A similar conclusion was drawn by Prado K et al. [40]. It should be pointed out that CSCs are a diverse population of cells with relatively high plasticity and a reversible phenotype. Altogether, it seems that the FDA-approved drugs may reduce the number of CSCs. However, an insufficient number of pre-clinical and clinical studies on CSCs in NSCLC patients make it impossible to evaluate whether the new targeted therapy may reduce the number of lung CSCs.

## 3. Lung Cancer Stem Cells

**Figure 2:**
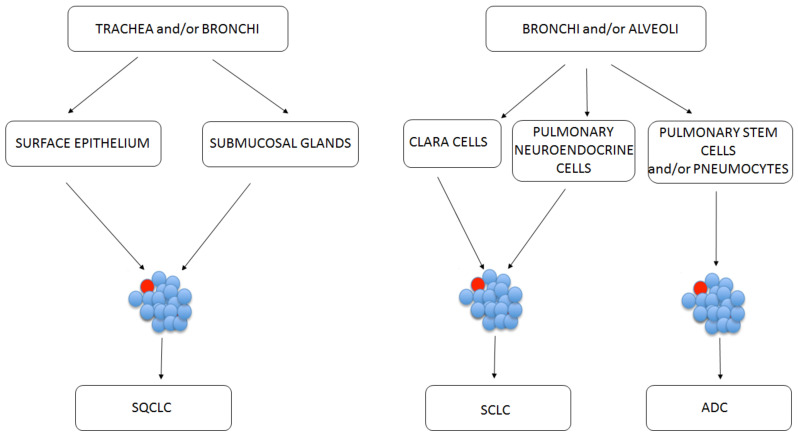
Possible CSC (marked red) initiation sites depending on the lung cancer subtype.

The origin of lung CSCs is now widely discussed. The most common hypothesis states they result from normal tissue-specific stem cells in their original tissues. Nevertheless, the identification of stem cell origin in the lungs presents a challenge, because the tracheal and bronchiolar epithelia are quiescent, having a low proliferative fraction [7]. Thus, in some simplification, the origin of lung CSCs has been traced back to cells on specific anatomical sites on lungs (Figure 2). The basal cells of proximal airway, such as trachea and bronchi, are linked with SQCLC showing stem-cell-like behavior [7]. Clara cells and pulmonary neuroendocrine cells are linked with SCLC showing stemness properties. ADC is linked with normal stem cells from the bronchoalveolar duct junction area [50].

Although the present knowledge on lung CSCs functions is restricted, a number of CSC markers have been proposed. CSCs are usually isolated due to the presence of specific molecules on their surface or in their interior. These are often markers belonging to CD (cluster of differentiation). Many studies have confirmed the presence of the following molecules on lung CSCs: CD133, CD44, CD90, epithelial cell adhesion molecule (EpCAM), C-X-C chemokine receptor type 4 (CXCR4) [51,52].

- EpCAM—transmembrane glycoprotein expressed in most human carcinomas; identified as a marker for carcinoma; can be attributed to its high expression on rapidly proliferating tumors of epithelial origin [52].
- CD133—a marker frequently used for identification of stem cells in both cancer and normal tissues. The process of CD133 transcription is regulated by five promoters, and promoter 5–P5 seems to play crucial role by CD133 expression in CSCs [53]. Some research has characterized CD133+ cells in NSCLCs [7,52]. For example, Eramo et al. showed that CD133 was present in a variable, but small number of NSCLCs, usually limited to <1% of cells [53]. CD133+ cells were capable in approximately 30% of cases to form tumor spheres in vitro when grown in serum-free medium; CD133+ cells derived from tumor spheres are capable to induce tumors when inoculated into immunodeficient mice with histological features similar to those of the original tumor [53]. Moreover, CD133+ positive cells display resistance to chemotherapy as a result of expressing high levels of ATP-binding cassette G2 [54].
- CD44—a transmembrane glycoprotein that binds hyaluronic acid, an abundant polysaccharide in stem cells. CD44 is responsible for various signaling functions (cell differentiation, survival, apoptosis, migration and proliferation). Some current studies revealed that CD44 plays a crucial role in CSC function such as self-renewal, resistance to apoptosis and niche preparation [7,55]. It has been shown that the mutations of p53 may be linked with up-regulation of CD44, leading to the promotion of CD44+ cells [56]. CD44+ cells demonstrate the ability to form spheroid bodies in vitro [57]. Additionally, cells with CD44 + phenotype are capable of forming a tumor mass in vivo in immunodeficient mice [51,58].
- CD90—a glycosylphosphatidylinositol-anchored glycoprotein is expressed mainly in white blood cells and is involved in cell–matrix and cell–cell interactions. Though, CD90 has been described as a marker for different types of CSCs, the potential role of CD90 as a marker for lung CSCs has not yet been fully described [7,49]. It has been reported that CSCs with co-expression of CD44 and CD90 could be detected in primary lung cell lines [56]. Up to date, the mutations that activate the CD90 expression are unknown. Studies performed on mice model suggest that the DNA methylation has a role in promoting CD90 expression. Serial xenotransplantation of EpCAM+ CD90+ cells in immunodeficient mice revealed a rapid growth of EpCAM+ cells in the subcutaneous lesion and a highly metastatic capacity of CD90+ cells in the lung [51].
- CXCR4—a chemokine receptor present on the surface of hematopoietic stem cells involved in trapping of these cells in the stem cell niches [59]. The CXCR4/CXCL12 pathway is responsible for tumor metastasis, progression, induction of angiogenesis, and resistance to apoptosis. Moreover, CXCR4 is presented on circulating tumor cells released from tumors into the peripheral blood, which induces their spread to CXCL12-positive distant sites [60]. The expression of CXCR4 is regulated by the Nuclear Respiratory Factor—NRF. NRF mutation may lead to the higher expression of CXCR4 [61]. CXCR4+ cells isolated from NSCLC lines were able to form the tumor spheres in vitro, had self-renewal capacity, demonstrated radiation resistance in vitro [62].

We identified lung CSCs exhibiting CD133, CD44, CD90, EpCAM, CXCR4 in lung cancer patients in our previous studies [63,64,65]. The rationale for the identification of CSCs according to some authors is that they seem to be more stable, guarantee resistance to systemic therapies and, in our view, are capable of bringing information for the modification of immune response in the site of the tumor [7,66].

As mentioned before, there is an insufficient number of pre-clinical and clinical studies on CSCs in NSCLC patients. However, patient-derived lung cancer organoids seem to be an interesting alternative. Patient-derived lung cancer organoids recreate the tissue architecture of primary lung tumors and maintain genomic alteration of primary tumors during long-term in vitro expansion [67]. The organoid culture method enables the in vitro CSCs’ multiplication by reflecting the complexity of tumor formation using primary cancer tissues and tumor xenografts. What is more, organoid culture allows functional analyses of CSCs, including their genetic engineering using CRISPR/Cas9-mediated genome editing [68]. Patient-derived organoids can be applicable to identify treatment resistance signatures of cancer stem cells in treated organoids. Unfortunately, the majority of cancer organoid models are limited to adenocarcinomas [69]. Thus far, less opportunities are currently available for squamous cell carcinomas for instance. However, as we broaden our understanding of tumor development and more, cancer stem cell markers are identified, organoids can only become a stronger research tool covering all types of cancer [69]. Thus, combined with other in vivo experiments, such as xenotransplantation assays of CSCs, organoid cultures of human CSCs have a high potential to advance our understanding of human cancer biology [70].

Another important contributing factor for CSCs is their function in causing treatment resistance. They are resistant to conventional radiotherapy and chemotherapy. It is thought to be related to their activation of different signaling pathways such as: Wnt (Wingless-type), Notch, and Hedgehog [71]. There is now mounting evidence that these pathways are deregulated and mutated in cancer and CSCs [72]. Aberrant Wnt signaling is found in many cancers, including NSCLC, especially ADC subtype [73,74]. In ADC, Wnt-reactive cells showed proliferative potential and progression, suggesting they have CSC characteristics [74]. A growing body of evidence supports the association of Notch signaling dysregulation with various types of malignancies, including NSCLC [75]. Notch signaling pathway play a role in stem cell maintenance in NSCLC; aberration in that pathway may result in increasing the number of CSCs resistant to platinum-based therapy [76]. It has been reported that increased Notch activity was associated with forming tumor spheres in vivo [77]. In the same study, Notch activity has been associated with a poorer prognosis in ADC patients, suggesting a potential role for inhibition of Notch activity as a new therapeutic approach for these patients [78]. In NSCLC, Hedgehog pathway is closely associated with CSCs [79]. Aberrant Hedgehog pathway is implicated in the maintenance of CSCs [80]. What is more, Hedgehog pathway is involved in tumor drug resistance in NSCLC, as cytotoxic chemotherapy, targeted therapies and radiotherapy [79].

## 4. Cancer Stem Cells and Tumor Microenvironment

CSCs are small numbers of cells that exist in the tumor microenvironment (TME) [45]. Lung cancer TME is composed of a various group of non-cancer cells, such as tumor-associated macrophages (TAMs stromal cells), regulatory T cells (Tregs), tumor-infiltrating lymphocytes (TILs), dendritic cells (DCs), natural killer (NK) cells, natural killer T (NKT), myeloid-derived suppressor cells (MDSCs), along with cancer cells: mature cancer cells and CSCs [46,80]. As yet, the complexity of the interactions between the cells in the immune TME has not been exhaustively described. Indeed, each cell involved, immune cell or tumor cell may influence the immunological behavior of the other cells, either distant or adjacent, within the TME. The mechanisms of such interactions include direct regulatory feedback, both paracrine and autologous, as well as co-inhibitory and co-stimulatory receptors through ligand engagement [81]. Cells are able to modify chemokine and cytokine secretion, with an imbalance between those with suppressive and activating immune functions. The source of intercellular communication is a complex network of cytokines, chemokines, growth factors, inflammatory mediators and enzymes [82]. In general, the suppressor function of the immune system predominates in TME. Some studies showed also that CSCs may activate mechanisms to circumvent a possible attack from the immune cells: loss of cancer antigen expression, initiation of oncolytic pathways, and promotion of immunosuppressive milieu [83]. Some studies demonstrated that CSCs deriving from different solid tumors, such as glioblastoma multiforme (GBM) and melanoma, release a variety of immunosuppressive cytokines, such as IL-13 and IL-10, transforming growth factor β1 (TGF-β), growth/differentiation factor 15 (GDF-15), prostaglandin E2 (PGE2), and galectin-3, that can render the TME protected from effector immune cells [84]. In particular, CSCs can induce the differentiation of MDCS or of Tregs through the release of TGF-β [85]. TME is an area that can regulate tumor development and self-renewal at the same time. CSCs can promote the expansion of the local vascular niche and local angiogenesis by releasing vascular endothelial growth factor (VEGF) [86]. TME surrounds and actively interacts with CSCs. It provides the ground to induce or recruit the differentiation of suppressive immune cells, including suppressive macrophages (M2 type) or Tregs [85,87]. Additionally, TAMs’ population increases the activity of transcription factors: Sox, Oct-4 and Nanog, which maintains the CSCs in the state of proliferation and self-renewal [88].

MDSCs represent the heterogeneous group of immature myeloid cells (precursors of macrophages, dendritic cells and granulocytes). In lung cancer, their recruitment occurs in pathological conditions through the secretion of appropriate cytokines by TME [89]. MDSCs show pro-angiogenic activity, induce the production of metalloproteinases and contribute to the formation of pre-metastatic niches that facilitate the colonization of cancer cells [90,91]. Reports from recent years indicate that MDSCs affect the expression of oncogenes in CSCs and induce their proliferation [89,90]. Furthermore, MDSCs can regulate CSCs by the secretion of pro-inflammatory cytokines: IL-1, IL-8, IL-6 [89,90].

TME elicits differentiation of the CD4+ T cells into different subsets of T cells, particularly suppressive Tregs, and T helper 17 (Th17) cells. Despite this, the exact role of the last cells in tumor immunity is still unclear depending on tumor stages and histological subtypes. Intriguingly, recent reports demonstrate that Tregs, under certain circumstances, express IL-17, which together with hypoxia plays a pivotal role in the regulation of CSCs [92]. Nonetheless, the interactions between CSCs and Tregs, which play an important immunosuppressive role in the TME, are still poorly understood.

The understanding of the immunological profile of CSCs and their interaction within TME has provoked the investigation of the immunological targeting of these cells (Table 3).

## 5. Liquid Biopsy

Tissue biopsy is still the ‘gold standard’ for diagnosis and biomarker testing in NSCLC [105]. However, the new method, liquid biopsy, was introduced if there is inappropriate material from tumor tissue at diagnosis. Inappropriate biopsy sampling/handling procedures should not be accepted by services as a reason to rely on peripheral blood examination. This liquid biopsy is an original and important material in lung cancer diagnosis and management. The idea to use body fluids and peripheral blood for oncological investigations tends to replace the invasive procedures and provide a more efficient monitoring of disease progression and therapeutic efficacy. Therefore, an increased use of liquid biopsies is also expected. This concept is especially important in the cause of lung cancer as the tumor is often difficult to obtain and may require a potentially harmful procedure and invasive. The International Association for the Study of Lung Cancer (IASLC) has stated that liquid biopsy methods have great potential to improve patient care, and immediate implementation in the clinic is justified in many therapeutic settings relevant to NSCLC [106]. Liquid biopsy has the potential to help manage NSCLC treatment throughout all stages of lung cancer: screening, minimal residual disease detection to guide adjuvant treatment, systemic treatment initiation and monitoring of response early detection of relapse and resistance genotyping [107]. Tissue biopsy offers only a piece of the tumor at a given location and time. It should be pointed out that liquid biopsy may have the potential to overcome both temporal and spatial tumor heterogeneity. Additionally, it can noninvasively explore the genetic landscape of a cancer taking into account many clones present at all metastatic areas and can follow subclonal evolution through multiple blood draws. However, we should be aware of the liquid biopsy limitations. More studies are needed to assess the liquid biopsy accuracy and its ability to identify various tumor types. It is not clear if the liquid biopsy provides a representative sampling of all genetic clones within a tumor or if there is a bias to specific subregions of the tumor. Considering that circulating tumor cells or DNA are relatively rare compared to the number of hematological molecules found in the blood sample, there are challenges to the detection ability of the test [106,108].

For appropriate molecular diagnosis, sampling regimens and liquid or tissue biopsies perfectly account for any clonal evolution leading to intra- and inter-tumor heterogeneity in multiple small primary tumors or multiple metastatic nodules from the same primary tumor. Nowadays, the inter- and intra-tumor heterogeneity are the main factors contributing to drug resistance and therapeutic failure [108]. Intra-tumor heterogeneity may have an impact on cancer therapy and biomarker discovery, especially in the era of targeted therapy [108]. However, if the tumor contains many branched events, indicating heterogeneity within the tumor, then even targeting the causative event may not have a significant effect on treatment outcome due to the low-frequency subpopulation carrying the branch resistance event, leading to subclonal selection and the acquisition of drug resistance, as observed with the mutation in *EGFR* in NSCLC [109]. Repeatable sampling of tumor genomes from liquid biopsies can help in identifying targetable molecular alterations within the tumor and nowadays is increasingly being used to monitor clonal evolution [110].

However, given the much lower overall sensitivity of the test, it is unlikely that a liquid biopsy will replace molecular examination of the tissue in the near future. Liquid biopsy is more likely to complete the tissue assessment in a cumulative approach, addressing the restriction associated with either subtype of testing material. What is more, test sensitivity still poses a big challenge. Considering that circulating tumor cells or circulating tumor DNA is relatively rare compared to the number of hematological molecules found in a peripheral blood sample, there are many challenges associated with detectability of the test [108,111]. In our own studies, we tried to detect circulating tumor cells and circulating CSCs using flow cytometry. Unfortunately, the number of cells was too low to perform a proper analysis [63,65,112]. Nowadays, the CellSearch test is the only test approved by FDA for CTC assessment. The international literature reported low CTC detection rate in NSCLC patients at the CellSearch analysis, which does not exceed 40% [111]. That is why another material obtained from NSCLC patients should be considered. Tumor-derived extracellular vesicles (TEVs) seem to be another promising material in body fluids. TEVs are unique particles that carry proteins and genetic information from parental cells [113]. They are released from various tumor cell types, which comprise exosomes and microvesicles originating from plasma membrane [114]. The TEVs have received a lot of attention during the past few years, mainly due to their increased prevalence and their promise as potential biomarkers to aid in the disease management [115,116]. The TEVs have been found to play various roles directly related to the disease progression and metastatic processes, also in NSCLC [117,118,119]. The TEVs also contribute to the suppression of antitumor immune responses by carrying different immunosuppressive particles [120]. While technical challenges arise, such as: isolation, enumeration, differentiation and molecular profiling of pure TEVs from the body fluid of cancer patients. It is difficult due to all the contaminants present, including proteins, protein aggregates, free nucleic acids, platelets and extracellular vesicles of different cellular origins [113].

Classically, the following material are listed as liquid biopsy: blood, serum, plasma, urine, pleural effusion [121]. A very useful method that fulfils the criteria of liquid biopsy is bronchoalveolar lavage (BAL) and fine needle aspiration (with an example of transbronchial needle aspiration guided by endobronchial ultrasound).

## 6. Endobronchial Ultrasound-Guided Trans-Bronchial Needle Aspiration (EBUS-TBNA)

In recent years, EBUS-TBNA has become a developed technique for NSCLC diagnosis and staging. It has been successfully introduced into daily clinical practice with many advantages, such as safe, cost-effective and minimally invasive approach, real-time image guidance, broad sampling capability, and rapid on-site evaluation [122,123,124].

**Figure 3:**
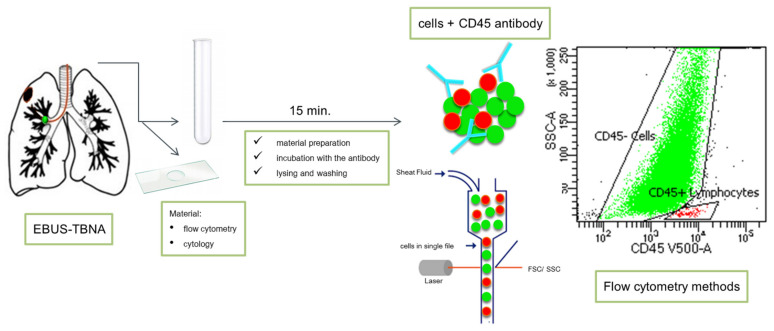
Determination of cells in EBUS/TBNA using flow cytometry methods. CD45 vs. SSC-A plot: Broad selection of cancer cells based on their SSC/CD45 properties (green: “CD45-cells” cancer cells; red: lymphocytes).

Based on our own experiments, we initially showed that the assessment of tumor cells in EBUS using the flow cytometry method is feasible (Figure 3). It relies on one staining with the CD45 antibody, specific for hematopoietic cells, and using the side scatter channel (SSC) parameter, which provides information about the internal complexity (i.e., granularity) of a cell.

The new therapies, among other immunotherapies with immune check point inhibitors (ICIs), require modern techniques for precise lung cancer diagnosis. When EBUS-TBNA is used as an initial diagnostic method after a CT scan in patients with suspected lung cancer limited to the thorax, it can provide an accurate nodal stage and diagnosis simultaneously [122]. A study by Tajarernmuang et al. conducted on a group of 120 patients suggests that findings in EBUS-TBNA samples can guide ICI therapy, with treatment results of PD-L1 expression comparable to histological specimens [125].

In our previous work, we assessed the phenotype of CSCs and examined the expression of PD-L1 on CSCs in metastatic lymph nodes (LNs) in NSCLC patients using flow cytometry. We described the presence of PD-L1 on putative lung CSCs: CD45- CD133+ EpCAM+ cells. A higher frequency putative lung CSCs was observed in patients with metastatic LNs than in patients without LN metastases. The presence of PD-L1+ CSCs in the metastatic LNs might suggest their immunogenic potential. Thus, flow cytometry analysis of the cells and immune molecules in metastatic LNs may be useful in “immunoscoring” before immunotherapy [63].

In our study, we analyzed the presence of immunosuppressive molecules: PD-L1, CD47, CD73, Fas and FasL on tumor cells and CSCs in LN aspirates and referred it to the lymphocyte subpopulation in peripheral blood. We found a higher frequency of tumor cells and CSCs with immunosuppressive molecules in metastatic LNs than in nonmetastatic. Interestingly, the expression of PD-L1 and CD47 was significantly higher on CSCs than on tumor cells [65]. We also analyzed the PD-L1+ positive CSCs in the context of T-cell repertoire and expression of immunomodulatory molecules in metastatic and non-metastatic LNs of NSCLC patients. We found that the percentage of PD-L1+ CSCs correlates with the percentage of CD4+ T cells and Tregs. Positive correlation between PD-L1+ CSCs and Tim3+ CD4+ T cells, as well as PD1+ CD4+ T cells, suggests that CSCs interact particularly with T cells. The frequency of PD-L1+ CSCs is associated with an altered T-cell frequency and phenotype indicating that CSCs can affect the immune system [64]. The higher percentage of PD-L1+ CSCs in patients with progressive disease may confirm their resistance to conventional therapy, suggesting that CSCs may be an interesting target complementary to immunotherapy [64].

## 7. Bronchoalveolar Lavage (BAL)

Bronchoalveolar lavage (BAL) is the recognized method of obtaining material from peripheral airways, which enables identification of the type of local immune response [126]. Taking into account the limited ability to assess tissue from the lung TME, BAL fluid examination seems to be a valuable alternative. A low number of lung cancer resections causes very low availability of TME examination. BAL is a relatively minimally invasive method and can replace TME analysis in the cases of the low availability of the appropriate fragment of the corresponding cancer tissue together with the surrounding tissue. In addition, it is possible to check the cytokine profile and fully assess the immune response in TME. The cytokine concentration and cellular pattern in BAL fluid, which can be performed during diagnostic bronchofiberoscopy, represent the alteration changes in TME. The nature of BAL fluid qualifies this material for flow cytometry analysis with a precise assessment of lymphoid cells phenotype and examination of surface and cytoplasmic molecules’ regulatory properties. BAL meets the diagnostic criteria in peripheral tumors and in disseminated malignancies in the lung [126]. The results of our previous studies have shown that the composition of BAL fluid effectively characterizes the local immune response in NSCLC patients [127,128]. BAL analysis may help in recognition of ‘hot’ immune response before immunotherapy [129,130]. Moreover, we hypothesized in our previous study that BALF from the ‘healthy’ lung, which is easier to obtain than tissue, may serve for the evaluation of individual natural profile of the anti-cancer response.

In our previous review, the literature was searched from the introduction of BAL to the diagnosis of lung diseases. We analyzed our previous original studies with the help of a bibliography and presented the usefulness of BAL in the diagnosis of the peripheral spread of neoplastic diseases and in the assessment of TME in lung cancer. Additionally, the article includes a commentary on the methodology of BALF analysis in lung cancer [131].

Exosomes are the new promising findings in BAL. These are small vesicles that originate from tumor cells and immune cells containing the antigenic and molecular information [132]. It is suspected that exosomes can modify the immune reaction in the neoplastic environment; therefore, their phenotype is investigated in different malignant tumors.

The assessment of BAL fluid may be of importance in the TME study of lung cancer in two aspects. The first aspect is for characterizing the immune response by analyzing immune cells and mediators, and the second is for the molecular characterization of cancer by analyzing free DNA and exosomes.

## 8. Conclusions

Understanding the biology of cancer stem cells (progenitor cells, CSCs) is one of the greatest challenges in basic science and clinical oncology. The presented directions and research results show that these cells are significantly associated with the development of solid tumors, such as lung cancer. ADC, which is recently more and more frequent and is now precisely recognized, also in terms of molecular changes, needs special attention. Identification of CSCs in this type of cancer with the use of markers discussed above may contribute to the designation of new therapy directions. Investigation of somatic mutation in normal tissues and its role in tumor progression and aging will provide a new insight into cancer treatment. Direct studies of mutation load, mutation signatures, clonal dynamics, and cellular phenotypes will provide a bridge from epidemiological discoveries to mechanistic insights into the earliest stages of cancer. A liquid biopsy may improve the qualification of lung cancer patients to targeted therapies or immunotherapies, through the identification of appropriate tumor-specific targets and biomarkers and to better define the predictors of the response to modern therapies.

## Figures and Tables

**Table 1:** Clinical trials leading to FDA approval of drugs for advanced NSCLC-targeted therapies.

Drug	Trial	Study Population	Study Intervention	Reference
EGFR mutation positive				
Osimertinib	FLAURA	Advanced untreated NSCLC, Central nervous system metastases allowed	Osimertinib vs. control (Gefitinib/Erlotinib)	[13]
Afatinib	LUX-Lung3, LUX-Lung 6	Advanced untreated NSCLC	Afatinib vs. chemotherapy	[14]
Erlotinib	EURTAC	Advanced untreated NSCLC	Erlotinib vs. chemotherapy	[15]
Dacomitinib	ARCHER 1050	Advanced untreated NSCLC	Dacomitinib vs. Gefitinib	[16]
Gefitinib		Advanced untreated patients;	Gefitinib vs. Carboplatin/Paclitaxel	[17]
Erlotinib + Ramucirumab	RELAY	Advanced untreated NSCLC	Erlotinib + Ramicriumab versus Erlotinib	[18]
ALK rearrangement positive				
Alectinib	ALEX	Advanced untreated NSCLC; Central nervous system metastases included	Alectinib vs. Crizotinib	[19]
Brigatinib	ALTA-1L	Advanced untreated NSCLC; Central nervous system metastases included	Brigatinib vs. Crizotinib	[20]
Ceritinib	ASCEND-4	Advanced untreated NSCLC; Central nervous system metastases included	Ceritinib vs. platinum + Pemetrexed	[21]
Crizotinib	PROFILE 1014	Advanced untreated non-SQCLC	Crizotinib vs. platinum + pemetrexed	[20]
ROS1 rearrangement positive				
Crizotinib		Advanced NSCLC	Phase I trial; no comparator	[22]
Ceritinib		Advanced NSCLC included central nervous system metastases	Phase II trial; no comparator	[23]
Entrectinib	ALK-372–001, STARTRK-1, STARTRK-2	Advanced NSCLC	Integrated analysis of three phase1/2 trials; no comparator	[24]
BRAF V600E mutation positive				
Dabrafenib/Trametinib		Advanced NSCLC; pretreated	Phase II; no comparator	[25]
MET Exon 14 Skipping mutation				
Crizotinib		Advanced NSCLC	Phase II; no comparator	[24]
Capmatinib	GEOMETRY mono-1	Advanced NSCLC	Phase II; no comparator	[24]
NTRK Gene fusion positive				
Larotrectinib		Any *TRK*-positive cancers (3 Lung tumors)	Phase I/II; no comparator	[25]
Entrectinib	STARTRK-1; STARTRK-2	Advanced NSCLC; pretreated	Phase I; no comparator	[24]
RET Rearrangement positive				
Selpercatinib/LOXO-292	LIBRETTO-001	Any *RET* rearranged tumor includes central nervous system metastases	Phase I; no comparator	[26]
Cabozantinib		Advanced NSCLC	Phase II; no comparator	[27]
Vandetanib		Advanced NSCLC	Phase II; no comparator	[23]

**Table 2:** Hallmarks of aging in lung cancer.

Feature	Effect	Reference
Genomic instability	The major cause of neoplasia, cancer initiation, progression, and impact the overall prognosis of the affected lung cancer patient	[41]
Inhibition of telomerase activity	Chromosome destabilization causes cellular senescence and death; in lung cancer telomere dysfunction promotes progression, metastasis and was associated with poor prognosis	[42,43]
Epigenetic mechanisms: (DNA hypermethylation, altered chromatin remodeling and histone modifications)	Established during differentiation, stably inherited and maintained through multiple rounds of cell division; deregulation of miRNAs is associated with early recurrence of lung cancer lesions	[42,44]
Mitochondrial DNA alteration	Plays a pivotal role in tumorigenesis; evasion of apoptosis	[42,45]
Intercellular communication	establishes a distinct tumor microenvironment (TME) with various stromal cell types to support growth, angiogenesis and invasion; altered communication of tumor cells to immune cells enable immune surveillance	[46]
Extracellular matrix (ECM) dysregulation	ECM actively undergoes dynamic remodeling during all stages of cancer progression; crosstalk between tumor cells and immune cells within primary and secondary sites is fundamental to ECM remodeling that feeds back to regulate tumor cell dormancy and outgrowth	[47]
Stem cell exhaustion	Cancer and aging are two possible endpoints of stem cells exposed to mutagenic hits, which will cause cell cycle arrest, and apoptosis or senescence. Through the acquisition of mutation and genetic or epigenetic alterations, normal stem cells can become CSCs	[48,49]

**Table 3:** Recent advances in targeting CSCs by immunotherapy. DCs—dendritic cells; CAR T cells—chimeric antigen receptor T cells; ID8-T—epithelial ovarian cancer cell line.

Type of Immunotherapy	Condition	Study Intervention	Reference
DCs vaccination	SQCLC, melanoma	ALDH^high^ CSC-pulsed DCs	[93]
DCs vaccination	Squamous cell cancer, melanoma	CSCs lysate-pulsed DCs	[94,95]
T-cell therapy	Colon cancer	CD8+ cytotoxic T-cells, specific for the CSCs antigen	[96]
T-cell therapy	Prostate cancer	CAR T-cells against EpCAM antigen	[97]
Virotherapy	Glioblastoma	Oncolytic adenovirus targeting CD133+ CSCs	[98]
Virotherapy	Ovarian cancer	Oncolytic vaccinia virus targeting ID8-T tumor model that harbors CSCs	[99]
Virotherapy	Hepatocellular carcinom	Oncolytic measles viruses: targeting CD133+ CSCs	[100]
Virotherapy	Breast cancer	Oncolytic vaccinia virus targeting ALDH^high^ CSCs	[101]
Combined therapy	Bladder cancer	CSCs vaccine combinated with anti-PD-1	[93]
Monoclonal antibody	Breast cancer	Anti-CD44 antibody	[102]
CSC-CAR T	Prostate	EpCAM-specific CAR T cell	[93]
Targeting signaling pathway	Lung cancer	Hedgedog pathway inhibitor	[103]
CSC-primed T cells	Lung cancer	CD8+ cytotoxic T-cells, ALDH^high^ specific CSCs	[104]
